# Community involvement in health services at Namayumba and Bobi health centres: A case study

**DOI:** 10.4102/phcfm.v6i1.613

**Published:** 2014-12-09

**Authors:** Jane F. Namatovu, Fred Ndoboli, Julius Kuule, Innocent Besigye

**Affiliations:** 1 College of Health Sciences, Department of Family Medicine, Makerere University, Uganda; 2 Department of Medicine, Gulu University, Uganda

## Abstract

**Background:**

Community involvement has been employed in the development of both vertical and horizontal health programmes. In Uganda, there is no empirical evidence on whether and how communities are involved in their health services.

**Aim and Setting:**

The aim of this study was to establish the existence of community involvement in health services and to identify its support mechanisms in Namayumba and Bobi health centres in Wakiso and Gulu districts, respectively.

**Methods:**

Participants were selected with the help of a community mobiliser. Key informants were selected purposively depending on their expertise and the roles played in their respective communities. The focus group discussions and key informant interviews were audio-recorded and transcribed verbatim. The transcripts were analysed manually for emerging themes and sub-themes.

**Results:**

Several themes emerged from the transcripts and we categorised them broadly into those that promote community involvement in health services and those that jeopardise it. Easy community mobilisation and several forms of community and health centre efforts promote community involvement, whilst lack of trust for health workers and poor communication downplay community involvement in their health services.

**Conclusion:**

Community involvement is low in health services in both Namayumba and Bobi health centres.

## Abstrait

**Participation de la communauté aux services de santé aux centres de Namayumba et Bobi: étude de cas**.

**Contexte:**

On a eu recours à la participation de la communauté pour développer des programmes de santé verticaux et horizontaux. En Uganda, il n'existe pas de données empiriques indiquant si et comment les communautés participent à leurs services de santé.

**Objectif et lieu:**

Le but de cette étude était de déterminer l'existence d'une participation de la communauté aux services de santé et d'identifier ses mécanismes de soutien aux centres de santé de Namayumba et de Bobi dans les districts de Wakiso et Gulu, respectivement.

**Méthodes:**

On a sélectionné les participants grâce à une personne qui a mobilisé la communauté. On a sélectionné exprès des informants clés selon leur expertise et leur rôle dans leurs communautés respectives. On a enregistré et transcrit textuellement les discussions du groupe et les entrevues des informants clés. On a analysé manuellement les transcriptions pour déterminer les thèmes et sous-thèmes émergents.

**Résultats:**

Plusieurs thèmes sont apparus dans les transcriptions et nous les avons classés en gros selon ceux qui encourage la participation de la communauté aux services de santé et ceux qui la compromettent. La mobilisation communautaire simple et plusieurs formes d'efforts communautaires et des centres de santé encouragent la participation de la communauté, tandis que le manque de confiance envers les professionnels de la santé et une mauvaise communication freinent la participation communautaire à ses services de santé.

**Conclusion:**

La participation communautaire est faible dans les services de santé des centres de santé de Namayumba et Bobi.

## Introduction

The global vision of achieving Health for All by the year 2000 through a Primary Health Care approach highlighted community participation and involvement as the lead supportive activity.^1^ Community involvement is essential for the emancipatory change which is central to community health; only communities which have the capacity to challenge, question and create change can make better health decisions that are relevant, useful and sustainable within the context of the daily lives of their members.^2^

Community involvement has been employed in the development of mental health promotion programmes and public health projects and is, therefore, relevant in both vertical and horizontal health programmes.^3, 4, 5, 6^ However, community involvement has been defined in different ways by various authors. Some have defined community involvement as a shift in emphasis from external agencies supplying health services, to the people of the community becoming active participants in their own health.^7^ Others have defined it as a typology of four processes of social change; conversion, mobilisation, allocation of resources and instruction, yet some authors think it should be conceptualised as contribution, organisation and empowerment of communities.^8, 9^ As result of these different ideologies, community involvement as a strategy to achieve health for all has been difficult to implement in most communities.^10^

### Social value

The World Health Organization framework for health promotion recognises that health is related to social, cultural and structural factors in addition to biological and psychological factors.^11^ This approach recommends changing the physical and social environments in order to facilitate lifestyle change and health services development for better health. This requires a proper and deeper understanding of the local knowledge, beliefs and norms for the implementation and sustainability of any interventions to improve the health of populations or communities, otherwise it remains an empty rhetoric.^12^ Community involvement is a viable approach towards the achievement of socially-acceptable health services.

The success of any technical assistance to the development of health services in the community depends on the way it is received by the community involved.^13, 14^ In most cases, the community is a passive recipient instead of being an active partner. As a result, efforts by governments and development partners to develop the health services in communities are misdirected.

### Scientific value

In Uganda, external agencies fund most of the community health activities. There is no established systematic way of involving communities in their health services. The establishment of village health teams in the early 1990s was an effort to involve communities in their health services. However, this approach has remained slow and is not well coordinated. The linkage with the formal health system and the community remains weak.^15^ The community members mostly participate by offering labour or other resources in the hope of getting some form of remuneration and/or incentive.^16^ There is no empirical evidence regarding whether and how communities participate in their health services in Uganda.

### Aim and objectives

The aim of this study was to establish the existence of community involvement in health services and to identify the available support mechanisms in the communities for the support of community involvement in health services in Gulu and Wakiso districts, using Bobi and Namayumba health centres, respectively, as case studies.

## Research methods and design

### Study design

This was a qualitative cross-sectional study using focus group discussions and key informant interviews. The participants for both the focus group discussions and key informant interviews were identified and selected with the help of a community mobiliser.

### Study population and sampling procedure

The focus group discussions comprised community members in the catchment area of the two health centres. Key informant interviewees included local community leaders and health centre staff. The participants for the key informant interviews were selected purposively depending on their expertise and the roles they play in the community and the health centre.

### Data collection

The authors and four research assistants were all trained in qualitative interviewing and were versed in the study objectives. The research assistants conducted the focus group discussions with the authors taking field notes. Two authors conducted all the key informant interviews for consistency. Both the focus group discussions and the key informant interviews were audio-taped using a voice recorder. The focus group discussions were conducted in the local languages, Luganda in Namayumba health centre and Luo in Bobi health centre. Each focus group discussion lasted about 90 minutes. The audio-recordings were transcribed verbatim by the interviewers and the interviews were then translated into English by the interviewers. The key informant interviews were conducted in English and each lasted 30 to 40 minutes. The interviews were then transcribed verbatim by the interviewers, after which the transcriptions from both focus group discussions and key informant interviews were validated separately against the audio-recordings.

### Data analysis

All the authors familiarised themselves with the data. The four authors met in May 2013, when they reviewed and analysed the transcripts manually for emerging themes and sub-themes using the inductive approach. Emerging themes were developed by studying the transcripts repeatedly and considering possible meanings and how these fitted with the developing themes. The authors met again in June 2013 to develop the key findings.

### Ethical considerations

The study received ethical approval from the school of medicine research and ethics committee Makerere University College of Health Sciences (reference number #REC REF 2012-145).

## Results

A total of eight focus group discussions and eight in-depth interviews was conducted – two focus group discussions and in-depth interviews each for men and two each for women in the catchment area of each health centre in the study (demographics provided in Table 1). The participants were all adults of 18 years and above. These were conducted from December 2012 to March 2013 when it was decided that the data collected were sufficient. The respondents were community members and local leaders; there were no clear differences seen between the views of these two groups.

**TABLE 1:** Participants in the in-depth interviews and focus group discussions.

Sampling method	Gender and age	Catchment area
**In-depth interviews Participant**		
P01	Male, 40 years	Bobi
P02	Female, 32 years	Namayumba
P03	Male, 50 years	Namayumba
P04	Female, 32 years	Namayumba
P05	Female, 30 years	Namayumba
P06	Male, 38 years	Bobi
**Focus group discussions**		
F01	Male, 29 years	Bobi
F02	Female, 40 years	Namayumba
F03	Female, 36 years	Namayumba
F04	Female, 42 years	Bobi
F05	Male, 45 years	Namayumba
F06	Female, 52 years	Namayumba

Several themes emerged from the transcripts and we categorised them broadly into those that promote community involvement in health services and those that jeopardise it.

### Themes that promote community involvement

#### Communities easy to mobilise

It was evident from both focus group discussions and in-depth interviews that communities in the catchment area of the two health centres respond very well to community mobilisation efforts.

#### Community efforts

There are various community efforts to promote health and involvement of communities in health services, both with regard to utilisation and development. These community efforts were led by individuals, for instance, a head teacher of a local primary school mobilising pupils and a leader of the local business community mobilising community members to do general cleaning at the health centre, as well as local leader initiatives exemplified by the monthly sanitation day in Wakiso district:

‘If the business community is mobilised, it does the work but local leadership is not as effective as the business leaders. The problem is leadership LCI, II and even III [*Local Councils I, II and III*] but for me I am doing my work.’ (In-depth interview, P06, 38 years, male, Bobi)

‘I am part of the community, I have my business community here which I can mobilise to come and help. Like one time here it was bush; I mobilised the business community, we came and dug, slashed the compound of the health centre.’ (In-depth interview, P01, 40 years, male, Bobi)

‘The head teacher of a local primary school in our community brings pupils to clean the health unit compound once in every term. This is done to teach pupils how to participate in community activities.’ (In-depth interview, P03, 50 years, male, Namayumba)

‘Wakiso district leaders established a monthly sanitation day in order to promote health in Wakiso district. On this day, the leaders mobilise communities to come and do general cleaning at the health centre and other public places.’ (In-depth interview, P04, 32 years, female, Namayumba)

#### Health centre efforts

**Peer-review sessions amongst health workers:** Health workers meet regularly and review their conduct. They give feedback to one another indicating where one performed well and where improvement is needed. This encourages personal reflection on the part of each of the staff members.

Involvement of local leaders in meetings:

‘Currently during health centre meetings, the chairperson … is invited as a community representative. At least, he is informed of the programmes running and the plans.’ (In-depth interview, P02, 32 years, female, Namayumba)

### Themes that jeopardise community involvement

#### Lack of trust

There was a strong feeling amongst the respondents regarding their lack of trust in health workers. This lack of trust jeopardises community involvement in health services at the two health centres:

‘People have tried to work/collaborate with the health centre. They see the vehicle which brings drugs coming and within a week, drugs are not there. This problem makes communities believe that the drugs are stolen.’ (In-depth interview, P05, 30 years, female, Namayumba)

‘Leaders should say the truth.’ (Focus group discussion, F02, 40 years, female, Namayumba)

‘Health workers tell you no medicine but when you go to their clinics, the medicines are found.’ (Focus group discussion, F01, 29 years, male, Bobi)

‘I got fed up with that health centre. The health worker sent me to buy medicine elsewhere, when I entered the drug shop, she was the same person who sold me the drugs.’ (Focus group discussion, F03, 36 years, female, Namayumba)

This lack of trust stems from personal interaction between health workers and community members, political promises and attitudes exhibited by health workers:

‘During political campaigns, aspiring candidates promise us that the health centre will have everything needed. Yet, we continue seeing no change and this creates a feeling that the health workers are not giving us the things.’ (Focus group discussion, F05, 45 years, male, Namayumba)

#### Poor communication

There is poor communication amongst community members, health workers and local leaders. Community members feel the health workers do not listen to them and are never willing to answer their questions and/or concerns:

‘When village health team members refer patients to the health centre for some blood tests, the health workers send us away saying that they are tired of removing blood.’ (Focus group discussion, F04, 42 years, female, Bobi)

In addition, there are some communication gaps between the formal system of village health teams and the health centre staff:

‘We send patients to the health centre for treatment and blood tests but they are not worked on and they lose morale.’ (Focus group discussion, F06, 52 years, female, Namayumba)

## Discussion

Respondents were positive about their involvement in their health services. The communities are also willing to get involved in their health services, but there is no systematic way of doing this. Community involvement mainly involves manual labour in the form of cleaning the health facility compound for the community members. This finding is similar to the experiences in Zimbabwe^17^ and has also been documented in other publications.^7, 9^ The Uganda government, through its decentralised system of governance, has promoted community involvement in the social services, including health. This has not achieved the required level of community involvement for appropriate action for health since the respondents still do not have any concrete ideas regarding know how to get involved in their own health issues. As a result, the community remains dissatisfied with the health services and the health workers, in turn, become frustrated since their efforts are not appreciated.

The community efforts to promote community involvement have emerged spontaneously through individual and group initiatives. It was evident from the responders that these initiatives arise from a feeling of ownership of these health facilities. There is no systematic approach, however, to community involvement in the two communities. This is because of poor leadership on the part of local authorities and health centre staff. It is, for example, assumed that participation of local leaders in the health facility meetings and other health facility activities, such as receiving of medicines and other supplies, constitutes community involvement.

Poor communication between the health facilities and the communities served is a source of mistrust, frustration and failure to effectively utilise the available health services, all of which result in poor health outcomes. This has also been compounded by opportunistic politicians who make unrealistic promises. These promises raise the expectations of the communities that cannot be met by the available resources at the health facilities. The health workers become victims of circumstance, which further worsens their frustrations. Health facilities have tried to listen to the community by instituting suggestion boxes. However, the communities think that they should be in charge of the suggestion box, as they are concerned that their grievances may be ignored since they are likely to have a direct effect on the health facility staff.

### Recommendations

There is a need to understand the community's perception of community involvement in health services. Innovative systematic approaches to both empower and involve communities in their health services should be explored. This will harmonise health facility efforts with the expectations of the community. Health professions training programmes at all levels should impart the competence of involving communities in their health affairs. Efforts should be made to build trust between health facility staff through proper communication and by making the community take charge of the suggestion boxes.

## Conclusion

Community involvement is low in the health services in both Namayumba and Bobi health centres. The support mechanisms for promoting community involvement available include: communities’ willingness to participate; feeling of ownership of the health facilities amongst some community members; efforts on the part of local leadership and health facility staff; health facility-community linkages through village health teams; and regular attendance of health facility meetings by local leaders.
